# Chromosome-level genome assembly of a cliff plant *Taihangia rupestris* var. *ciliata* provides insights into its adaptation and demographic history

**DOI:** 10.1186/s12870-024-05322-y

**Published:** 2024-06-25

**Authors:** Wei-Guo Li, Yuan-Yuan Li, Chuan-Kun Zheng, Zhi-Zhong Li

**Affiliations:** 1 School of Resource and Environment, Henan Polytechnic University, Jiaozuo, Henan, 454000 China; 2 Wuhan Botanical Garden, Chinese Academy of Sciences, Wuhan, 430074 China

**Keywords:** Cliff plant, Harsh habitat, Taihang Mountains, Colurieae, Ecological adaptation

## Abstract

**Background:**

Cliffs are recognized as one of the most challenging environments for plants, characterized by harsh conditions such as drought, infertile soil, and steep terrain. However, they surprisingly host ancient and diverse plant communities and play a crucial role in protecting biodiversity. The Taihang Mountains, which act as a natural boundary in eastern China, support a rich variety of plant species, including many unique to cliff habitats. However, it is little known how cliff plants adapt to harsh habitats and the demographic history in this region.

**Results:**

To better understand the demographic history and adaptation of cliff plants in this area, we analyzed the chromosome-level genome of a representative cliff plant, *T*. *rupestris* var. *ciliata*, which has a genome size of 769.5 Mb, with a scaffold N50 of 104.92 Mb. The rapid expansion of transposable elements may have contributed to the increasing genome and its ability to adapt to unique and challenging cliff habitats. Comparative analysis of the genome evolution between *Taihangia* and non-cliff plants in Rosaceae revealed a significant expansion of gene families associated with oxidative phosphorylation, which is likely a response to the abiotic stresses faced by cliff plants. This expansion may explain the long-term adaptation of *Taihangia* to harsh cliff environments. The effective population size of the two varieties has continuously decreased due to climatic fluctuations during the Quaternary period. Furthermore, significant differences in gene expression between the two varieties may explain the varied leaf phenotypes and adaptations to harsh conditions in different natural distributions.

**Conclusion:**

Our study highlights the extraordinary adaptation of *T*. *rupestris* var. *ciliata*, shedding light on the evolution of cliff plants worldwide.

**Supplementary Information:**

The online version contains supplementary material available at 10.1186/s12870-024-05322-y.

## Background

Cliffs, known for their harsh conditions, pose significant challenges for plant survival. These extreme environmental factors, including drought, limited soil nutrition, and steep terrain, have shaped plants to develop extraordinary adaptations [1]. The formation of cliffs is often linked to ancient geological events, such as the rapid uplift of mountains during the Pleistocene, which led to the creation of these imposing vertical rock formations [2, 3]. As a result, the complex topography has fragmented large plant populations into numerous small groups due to geographic isolation [4, 5]. The absence of competitive exclusion, limited disturbance, and temperature buffering have allowed glacial relicts to persist in these isolated habitats [6]. Furthermore, the fluctuations in ancient climates have allowed lithophyte populations to occupy new ecological niches. The spatial and environmental heterogeneity among cliffs has been shown to drive the differentiation of lithophyte populations and even accelerate ecological speciation [5, 7–9]. Consequently, cliffs harbor exceptional ancient and endemic plant species, which are crucial in preserving biodiversity [10–12].

The Taihang Mountains, located in eastern China, serve as a natural boundary and stretch from southwest to northeast in the northern part of the country (approximately 36–40°N, 112–115°E). One notable characteristic of this region is the higher elevation in the North compared to the South [13]. The geological history of the Taihang Mountains dates back 2.5 billion years [5] and experienced significant uplift during the late Pleistocene, resulting in the formation of even taller mountains and deeper valleys within the plateau [14, 15]. The complex topography, combined with climate fluctuations, has contributed to the development of a diverse range of plant species, particularly cliff plants (such as *Opisthopappus* [16]; *Oresitrophe* [17]; *Taihangia* [7]), which are endemic to this region. Additionally, it is believed that the climatic changes and geographical isolation between the northern and southern parts of the Taihang Mountains have played a crucial role in the process of ecological speciation in this area [7, 17]. Recent studies have focused on various aspects of cliff plants in this region, including population genetics [7, 16], responses to stress in terms of growth and phenotype [9, 14], and reproductive ecology [18]. The case of *Opisthopappus* provides evidence that the rapid uplift of the Taihang Mountains during the Pleistocene resulted in local environmental and ecological heterogeneity, leading to the spatial and temporal isolation of two closely related sister species. Furthermore, climate fluctuations played a significant role in driving the diversification of these two species [5]. Despite these findings, there is a lack of comprehensive research on the biogeographic history of rare cliff plants and the underlying molecular mechanisms of adaptive speciation at the genomic level. This knowledge gap poses a challenge to effectively protect the endemic cliff plants in this ecologically significant area.

*Taihangia rupestris* Yu & Li, a rare perennial herb, exhibits a narrow and sporadic distribution pattern, inhabiting small crevices on vertical cliff faces within the latitude range of 35º27ʹ to 36º56ʹ in the Taihang Mountains [7, 19]. *T*. *rupestris* has been classified into two varieties, namely, *T*. *rupestris* var. *rupestris* and var. *ciliata*, based on differences in leaf shape and trichome density. The former, predominantly found in the northern region, possesses ovate or ovate-elliptic leaves with lobate serrations, while the latter, solely recorded in the southern region, exhibits a heart-shaped leaf blade with abundant and deep leaf margin serrations, suggesting their adaptation to the spatial variation in temperature and precipitation from north to south [14]. Regarding reproduction, *T*. *rupestris* primarily relies on vegetative propagation by forming short rhizomes along crevices on vertical cliff faces [20]. Consequently, the species faces challenges in terms of low seed germination rate and limited seed dispersal, impeding the natural regeneration of its populations [21]. Over the past few decades, the number of individuals, population sizes, and distribution ranges of *T*. *rupestris* has steadily declined, leading to its inclusion as a Grade II protected plant in China [22]. Previous research has provided evidence that the divergence of the two varieties occurred during the Pleistocene [23]. Genetic diversity was found to be high at the species level, with most of the variation observed within populations, suggesting an ancient origin and a unique reproductive system [7]. However, it remains unclear whether the population size of the two varieties fluctuated in response to climate changes during the Quaternary period. Additionally, the molecular mechanisms underlying the adaptability of leaf phenotype to habitat heterogeneity at the genomic level are still unknown.

In this study, we present a comprehensive genomic analysis of the cliff-dwelling plant *T*. *rupestris* var. *ciliata* at the chromosomal level. Our study focuses on two main objectives: 1) examining the evolutionary history of the two varieties in the Taihang Mountains and the influence of post-Pleistocene mountain uplift on their distribution and speciation; 2) investigating the potential molecular mechanisms underlying the differentiation of leaf phenotypes between the two varieties using RNA-seq data. The findings of this study will not only advance our understanding of the demographic history and adaptive processes of *T*. *rupestris*, but also provide valuable insights into the evolution of cliff-dwelling plants on a global scale.

## Results

### Genome assembly, annotation, and repetitive content

**Fig. 1:**
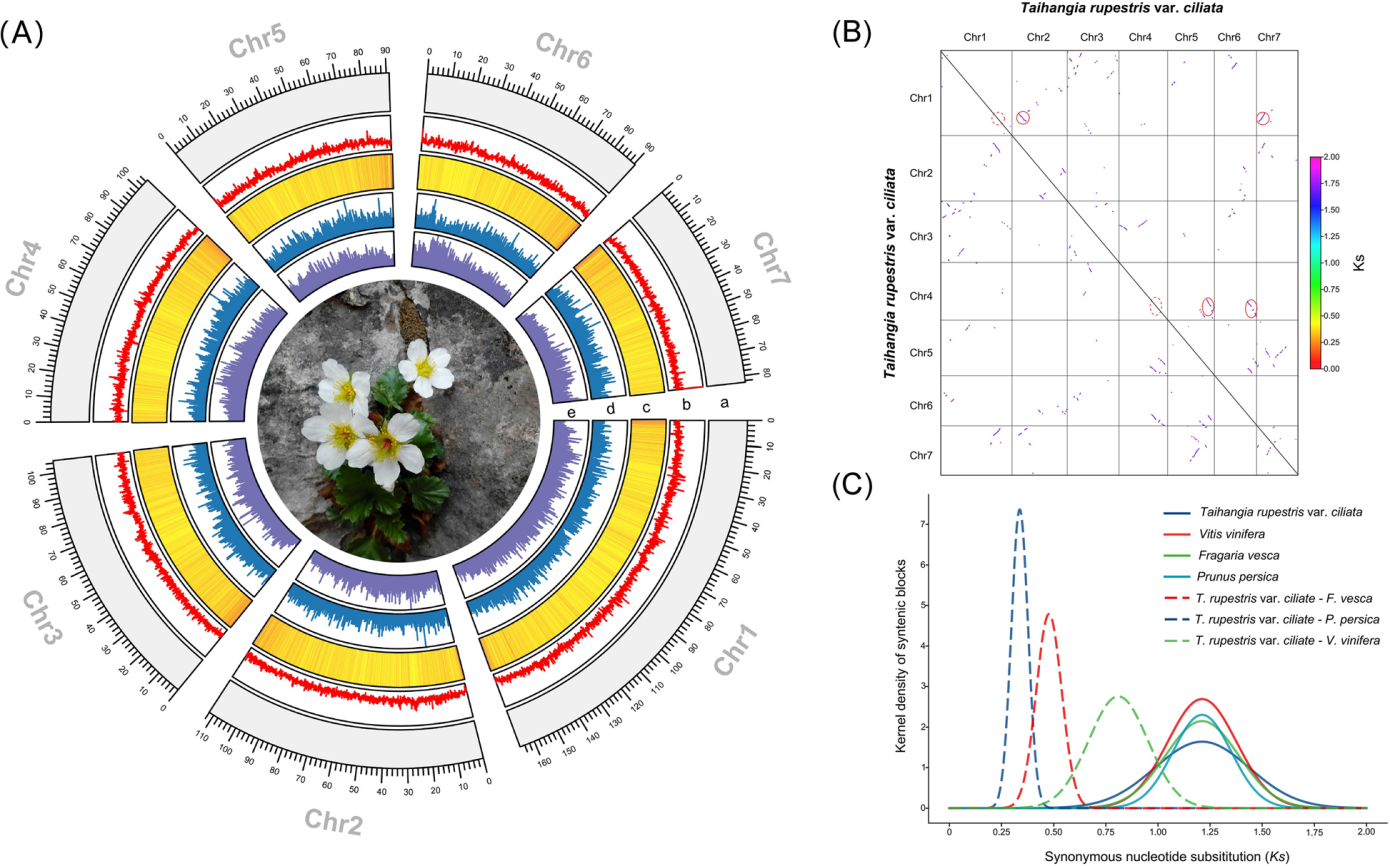
Characterization and whole genome duplication of the *Taihangia rupestris* var. *ciliata* genome. **A** Circos map of the *T. rupestris* var. *ciliata* genome. (**a**) pseudochromosome length; (**b**) GC content; (**c**) gene density; (**d**) LTR_*Gypsy* density; (**e**) LTR_*Copia* density. **B** Syntenic blocks within *T. rupestris* var. *ciliata*; **C***Ks* distributions of orthologous and paralogous genes among *T. rupestris* var. *ciliata*, *V. vinifera*, *F. vesca* and *P. persica*

Based on the analysis of 17-mer frequency, we estimated that the genome size of *T*. *rupestris* var. *ciliata* is approximately 863.27 Mb, with a heterozygosity rate of 0.89% (Fig. S1). For the initial assembly, approximately 27.9 Gb of sequencing data (~ 32 ×) were assembled into high-quality contigs, resulting in a contig-level genome assembly of around 769.4 Mb with an N50 of 17.87 Mb. After incorporating Hi-C data, approximately 752.24 Mb (~ 97.77%) of the assembly was successfully anchored onto seven pseudochromosomes (Fig. S2), with sizes ranging from 82.67 Mb to 169.97 Mb (Table S1; Fig. 1). This integration improved the N50 value to 104.92 Mb. Regarding the quality assessment of the genome assembly, we obtained positive results from three aspects: 1) CEGMA and BUSCO analysis revealed that 95.16% and 98.3% of the complete category, respectively, were identified using the embryophyta_odb10 database (Tables 1, S2, S3); 2) the assembly was evaluated to have a QV value of 46.99, indicating accuracy of over 99.99%; 3) 93.28% to 97.13% of RNA-seq data from various tissues and 99.52% of WGS reads from SSM2 were successfully mapped to the assembly (Tables 1, S4, S5). These results collectively demonstrate that our genome assembly of *T*. *rupestris* var. *ciliata* is highly consistent and complete. Additionally, we successfully predicted 36,300 PCGs, with 90.65% of them having functional annotations in public protein databases (Tables S6, S7). Additionally, 591 microRNAs, 1584 transfer RNAs, 7513 ribosomal RNAs, and 2292 small nuclear RNAs were predicted in the assembly (Table S8).

**Table 1:** Information of genome assembly and annotation of *Taihangia rupestris *var.* ciliata*

	**Results**
**Assembly feature**	
Total length of contigs (Mbp)	769.4
N50 of contigs (bp)	17.87
Number of contigs	307
Longest contigs (Mbp)	49.19
Total length of scaffolds (Mbp)	769.4
N50 of scaffolds (bp)	104.92
Number of scaffolds	239
Longest scaffolds (Mbp)	165.97
Hi-C anchor ratio (%)	97.77%
Core Eukaryotic Genes Mapping Approach(CEGMA) evaluation	97.18%
Benchmarking Universal Single-Copy Orthologs (BUSCO) evaluation	98.80%
RNA-Seq evaluation	92.69%~97.13%
Consensus quality (QV)	46.99
**Genome annotation**	
Percentage of repeat content (%)	70.57
No. of predicted protein-coding genes	36,300
No. of genes annotated to public database	32,906

### Genome evolution

**Fig. 2:**
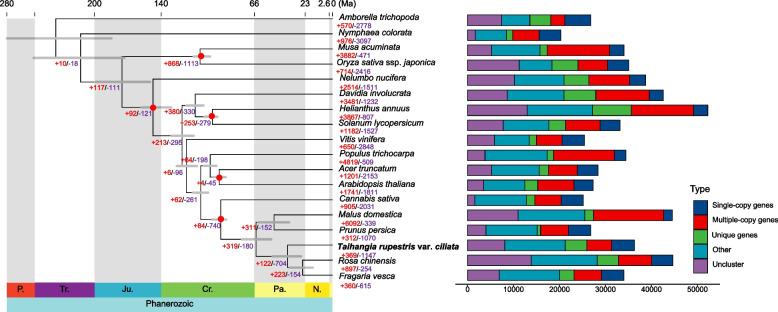
Phylogenetic tree of 18 plant species and the evolution of gene families. Read circles indicating the calibrating points

609,809 PCGs from 18 species were classified into 37,677 gene families, including 6,742 common gene families and 4,701 gene families unique to *T*. *rupestris* var. *ciliata* (Fig. 2). Among these, 423 single-copy genes were identified and utilized to reconstruct the phylogenetic tree. The phylogenetic analysis revealed that *T*. *rupestris* var. *ciliata* is closely related to *R*. *chinensis* and *F*. *vesca*, forming a monophyletic clade with *M*. *domestica* and *P*. *persica* within the Rosaceae family. The divergence time between Rosaceae and *Cannabis sativa* was estimated to be around 94 Ma, while *T*. *rupestris* var. *ciliata* diverged from the common ancestor of *R*. *chinensis* and *F*. *vesca* at approximately 38 Ma (Fig. 2). Furthermore, we identified 369 expanded gene families and 1,147 contracted gene families in *T*. *rupestris* var. *ciliata* (Fig. 2). Among these, 31 gene families consisting of 372 genes exhibited significant expansion (*P*-value < 0.05) and were primarily enriched in "metabolic process," including "peptide metabolic process (GO:0006518)," "translation (GO:0006412)," and "Oxidative phosphorylation (ko00190)" (Figs. S3 and S4).

**Fig. 3:**
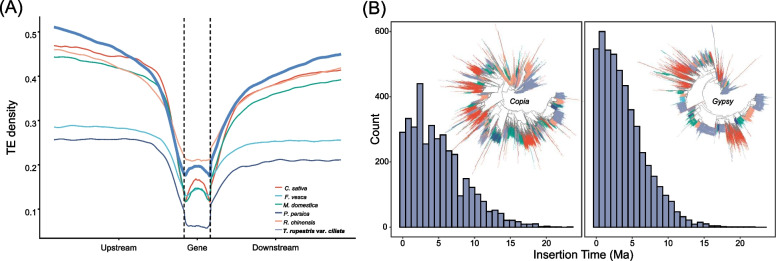
Comparison of transposon content in cliff and non-cliff plants in Rosaceae. **A** Comparison of TE density around the closest genes between species. **B** Insertion time of LTR-RTs in *T. rupestris* var. *ciliata* and the phylogeny of LTR-RTs in *T. rupestris* var. *ciliata*. (**a**) Insertion time and phylogeny of *Copia* superfamily; (**b**) Insertion time and phylogeny of *Gypsy* superfamily

By integrating homology-based and de novo approaches, we identified a high proportion of TEs in the genome assembly of *T*. *rupestris* var. *ciliata*, with nearly 70.57% of the assembly consisting of TEs, primarily long terminal repeats (LTRs) (Table S9). In comparison, other non-cliff Rosaceae species exhibited lower levels of TE content, such as 33.08% in *F*. *vesca*, 32.87% in *P*. *persica*, 56.32% in *R*. *chinensis*, 57.1% in *M*. *domestica*, and 68.5% in *C*. *sativa* (Table S10). Additionally, we observed higher TE density in the upstream and downstream regions of genes in *Taihangia* compared to other non-cliff species (Fig. 3).The constant expansion of intact LTR-RTs in *T*. *rupestris* var. *ciliata* over the past ten million years, with a notable burst in the last three million years (*Copia*: ~ 3 Ma; *Gypsy*: ~ 1 Ma; Fig. 3).

Based on the analysis of *Ks* distribution and syntenic relationships, we observed a single polyploidization event in *T*. *rupestris* var. *ciliata* (Fig. 1). This event is supported by the presence of a sole peak in the *Ks* distribution (*Ks* =  ~ 1.24) shared among *T*. *rupestris* var. *ciliata*, three Rosaceae species, and *V*. *vinifera*. Dot plots of paralogs within *T*. *rupestris* var. *ciliata* exhibited a 1:3 ratio (Fig. 1). In contrast, orthologs between *T*. *rupestris* var. *ciliata* and *V*. *vinifera* showed a 3:3 ratio (Fig. S5), indicating an ancient whole-genome triplication (γWGT) event in *T*. *rupestris* var. *ciliata*.

### Demographic history of Taihangia rupestris

**Fig. 4:**
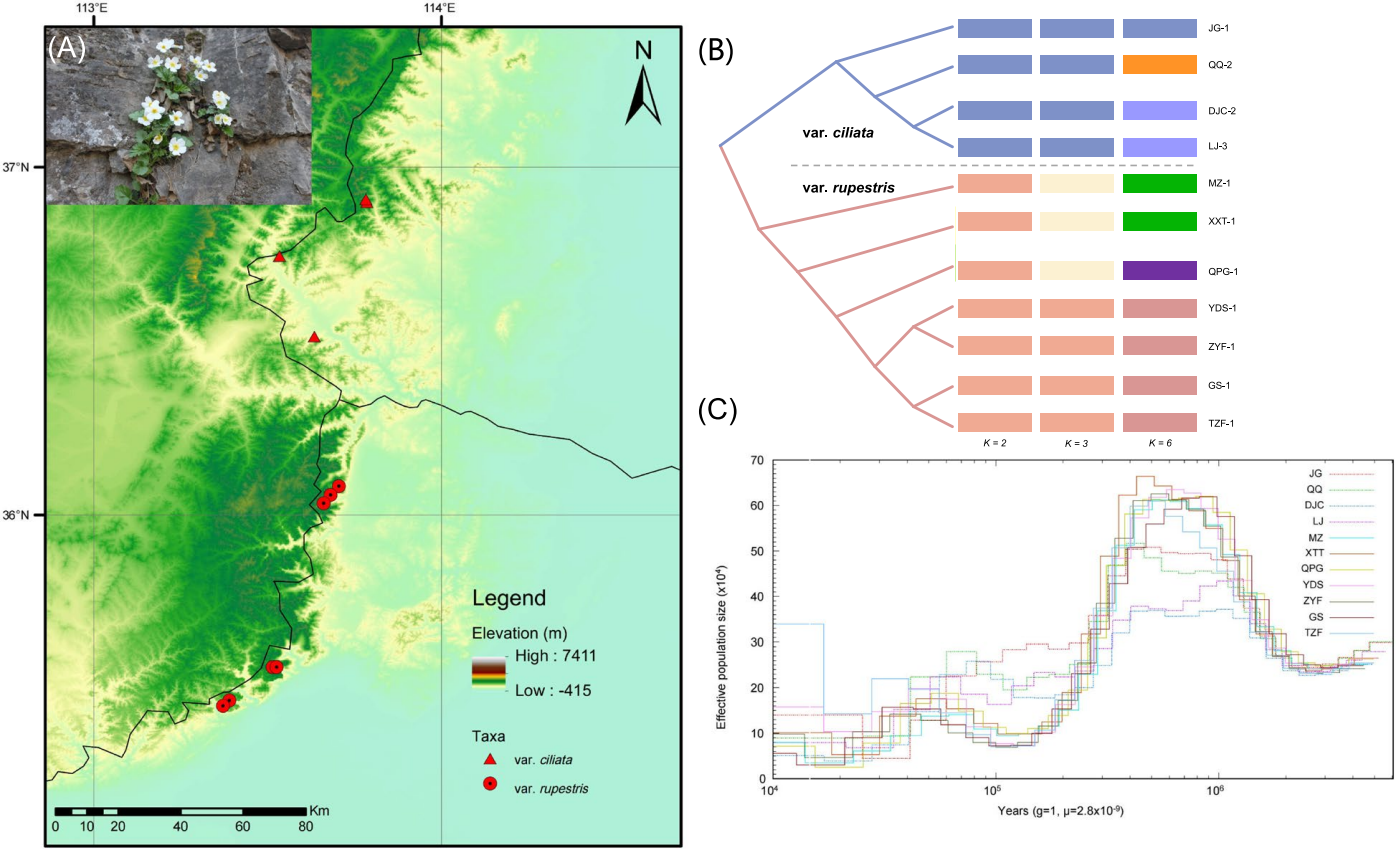
Population structure and demographic histories of *T. rupestris*. **A** Map showing the distribution of the 11 resequenced populations. **B** Phylogenetic relationship and population structure of the resequenced samples. **C** Demographic histories of *T. rupestris*

Phylogenetic and population structure analyses revealed two distinct groups corresponding to the two varieties of *T*. *rupestris* across Taihang Mountain (Fig. 4). The cross-validation (CV) from the Admixture analysis confirmed the existence of six genetic clusters, consistent with the geographic distribution of each population (Fig. 4; Table S11), indicating significant genetic differentiation within the varieties due to long-term spatiotemporal heterogeneity.

The PSMC analysis revealed a similar demographic history for all populations of the two varieties. During the Naynayxungla Glaciation (NG, 0.8–0.50 Ma) period in China, there was a sharp decline in effective population size (Fig. 4).

### Comparative transcriptome analysis of leaves

**Fig. 5:**
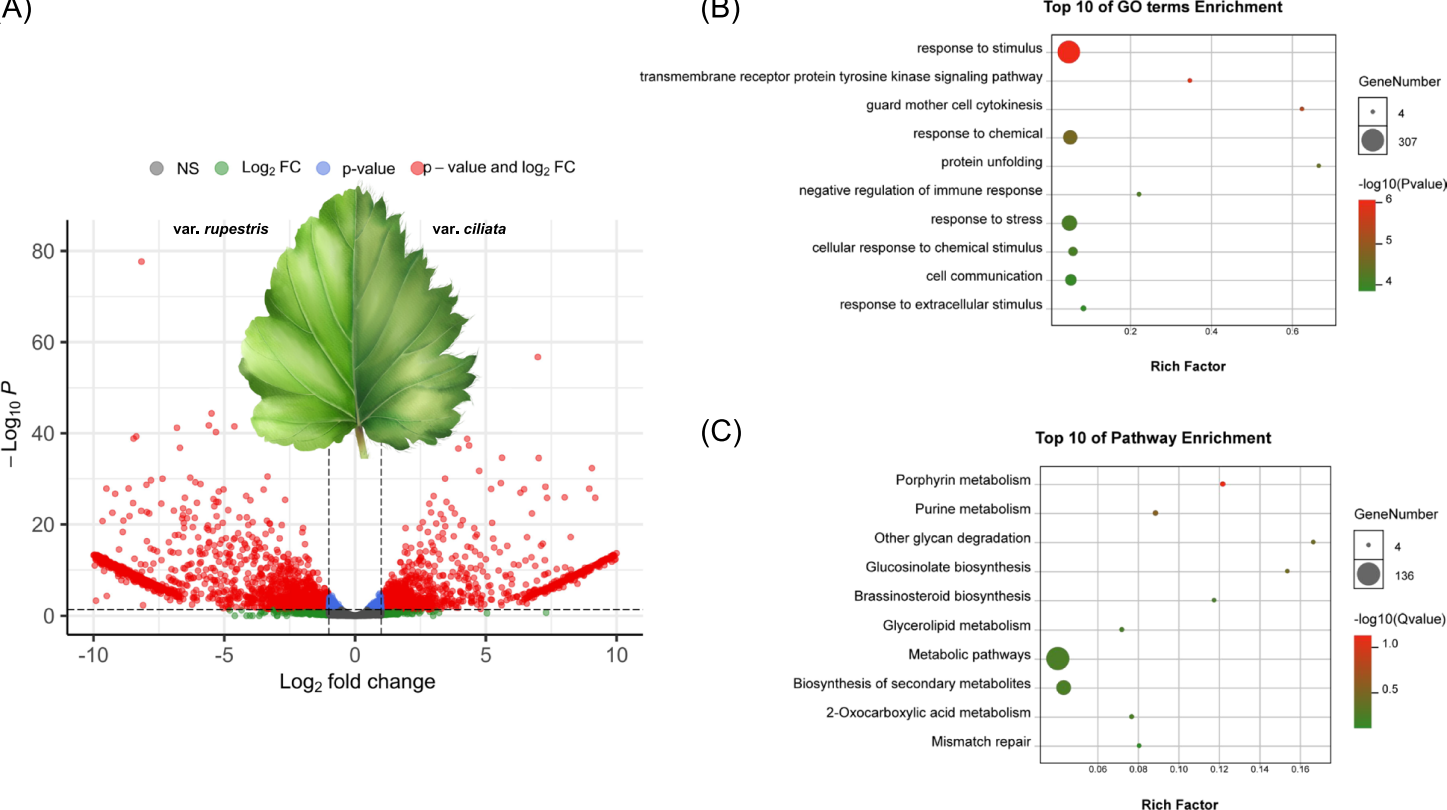
Overview of differentially expressed genes (DEGs) between two varieties of *T. rupestris*. **A** Volcano plot of the DEGs between two varieties of *T. rupestris*. **B** Top 10 GO terms enrichment of up-regulated DEGs. **C** KEGG pathways enrichment of up-regulated DEGs

Although *Taihangia* is narrowly distributed in the Taihang Mountains, the two varieties, var. *ciliata* and var. *rupestris*, exhibit contrasting habitats and climatic conditions. var. *ciliata* is predominantly found in semiarid zones characterized by lower temperatures and precipitation, whereas var. *rupestris* thrives in warm temperate semi-humid regions [14]. We conducted a comparative transcriptome analysis of their leaves to unravel the molecular mechanisms underlying the differentiation and variation between these two Taihangia varieties in response to different environments. Our analysis revealed that a total of 1342 genes exhibited significantly higher expression levels (up-regulated) in var. *ciliata* compared to var. *rupestris* (*P*-value < 0.05 & logFC > 1), whereas 2042 genes displayed lower expression levels (down-regulated) in var. *ciliata* in comparison to var. *rupestris* (*P*-value < 0.05 & logFC < -1; Fig. 5). According to the GO and KEGG enrichment analyses, the up-regulated genes in *T*. *rupestris* var. *ciliata* were primarily associated with stress response pathways (Fig. 5). These pathways included “response to stimulus” (GO:0050896), “transmembrane receptor protein tyrosine kinase signaling pathway” (GO:0007169), “Porphyrin metabolism” (ko00860), and “Purine metabolism” (ko00230).

## Discussion

In this study, we assembled the high-quality genome of the cliff plant *T*. *rupestris* var. *ciliata* at chromosome level. The genome size was approximately 769.4 Mb, which is notably larger than other non-cliff Rosaceae species, such as *F*. *vesca* (~ 211 Mb), *R*. *chinensis* (~ 518 Mb), and *P*. *persica* (~ 227 Mb) [24–26]. The eruption of transposable elements (TEs) in the genome of plants can have significant implications for their genome size and adaptation to different environments [27, 28]. In our study, compared to other non-cliff Rosaceae species, the cliff plant *T. rupestris* var. *ciliata* showed higher levels of TE content and TE density in the upstream and downstream regions of genes, these findings suggest that the accumulation of TEs may play a crucial role in adapting *T. rupestris* var. *ciliata* to cliff habitats and contribute to the expansion of its genome size. In addition, the constant expansion of intact LTR-RTs in *T*. *rupestris* var. *ciliata* was burst in the last three million years (Fig. 3), aligns with the divergence time of the two varieties (~ 1.5 Ma [23]). This indicates that recent LTR-RT evolution may have played a role in driving speciation in *Taihangia*, which is consistent with findings in other flowering plants such as *Oryza* [27] and *Arachis* [28]. Notably, LTR-RTs have been shown to respond to various environmental stresses, including heat [29], cold [30], and drought [31], which may account for the significantly higher number of intact LTR-RTs, particularly the *Gypsy* element, in *Taihangia* compared to other non-cliff plants in this study.

Gene family evolution analyses found that 31 gene family were significantly expansion which mainly enriched in "Oxidative phosphorylation (ko00190)". Oxidative phosphorylation, known for its role in plant response to abiotic stresses such as drought [32], likely plays a crucial role in *T*. *rupestris* var. *ciliata*'s adaptation to the harsh conditions of its cliff habitat. Compared to other non-cliff Rosaceae species, *Taihangia* has been reported to face challenges related to drought and nutrient deprivation, which are considered significant limiting factors for its survival in the cliff environment [14, 23]. The significant expansion of genes related to oxidative phosphorylation provides insights into the adaptive mechanisms of *Taihangia* in harsh habitats.Similar to other Rosaceae species [24, 26], only an γWGT event was identified in *T. rupestris* var. *ciliata*, although gene duplication resulting from WGD may play a role in facilitating plant adaptation to hash environments, selective retention of genes crucial for species survival is evident in *T*. *rupestris* var. *ciliata* [33], as supported by our functional enrichment analysis of WGT-related genes (Figs. S6 and S7). Notably, these genes were significantly enriched in "oxidoreductase activity, acting on NAD(P)H (GO:0016651)", "transferase activity, transferring phosphorus-containing groups (GO:0016772)", and "Plant hormone signal transduction (ko04075)".

Similar to previous study, two distinct clades corresponding to the two varieties of *T. rupestris* across Taihang Mountain was revealed by population genomic analyses. Also, a sharp decline in effective population size was displayed during the Naynayxungla Glaciation period, despite the absence of major Quaternary glaciations, Northern China, including Taihang Mountain, experienced significant climatic oscillations [4]. These climatic changes, influenced by Pleistocene glaciation, resulted in a transition from arid to cooler temperatures in most areas of Northern China, which may have posed challenges to the survival of plant species adapted to warm and humid environments [34]. Growing evidence suggests that mountainous regions have acted as refugia for plant lineages during glacial periods, playing a crucial role in the preservation of biodiversity in Northern China [35]. Compared to var. *rupestris*, the var. *ciliata* displayed a more moderate change of *Ne*, with populations of DJC and LJ being the most flat (Fig. 4). Located at the boundary between the Loess Plateau and the North China Plain, the northern Taihang Mountains, where var. *ciliata* is distributed, are considered important refugia during the NG period. Following the NG period, as the climate became warmer, all var. *rupestris* populations increased by approximately 0.1 Ma, while four var. *ciliata* populations continued to decline (Fig. 4). This suggests that var. *rupestris* populations could persist in these areas through in situ survival. Consistent with our findings, the studied populations of both varieties did not show significant expansion after the Last Glacial Maximum (LGM), except for the TZF population, which can be attributed to factors such as limited seed dispersal and the absence of suitable habitats [20, 21]. Given the limited sampling in our study, a more extensive sampling across diverse populations is necessary to gain a comprehensive understanding of the population dynamics and evolutionary processes of this endemic cliff plant in the Taihang Mountains.

The significant differences in gene expression profiles related to stimulus response between the two varieties may have played a role in their differentiation and adaptation to heterogeneous environments. This finding aligns with previous studies on the endemic cliff plant *Opisthopappus* in the Taihang Mountains, highlighting the importance of stress response mechanisms in adaptation [5].

Interestingly, we identified several up-regulated DEGs in var. *ciliata* that are involved in the response to water stress. These include aquaporin PIP (*evm.model.Chr5.4622*), abscisic acid-insentive 5-protein 7 (*evm.model.Chr3.6486*), and LRR receptor-like serine/threonine-protein kinase ERL1 (*evm.model.Chr7.155*). These genes play crucial roles in modulating water stress tolerance in plants, suggesting that var. *ciliata* may have enhanced adaptation to arid environments. Additionally, we found significant differential expression of genes related to stress tolerance in var. *ciliata*, such as Trehalose-6-phosphate synthase (*evm.model.Chr2.5495*), YTH Domain Protein ECT2 (*evm.model.Chr2.6228*), and Trihelix transcription factor DF1 (*evm.model.Chr3.1343*). Apart from their established roles in stress response, these genes are known to regulate leaf morphology in other plant species. For example, in *Arabidopsis*, *AtTPS6* controls leaf cellular morphogenesis, including trichome branching and leaf serration [36]. The higher expression of these genes in var. *ciliata* may contribute to the distinct leaf characteristics observed, such as deep serrations in the leaf margin [37–39]. Overall, our findings indicate that the DEGs identified in this study play a crucial role in the differentiation and adaptation of the two *Taihangia* varieties to their heterogeneous habitats. By regulating gene expression, these genes likely contribute to the ability of *Taihangia* to thrive in challenging cliff environments.

## Methods

### Genome sequencing

In 2018, healthy individuals of *Taihangia rupestris* var. *ciliata* (2n = 14) were collected from Sanshimu, Shanxi, China (36º40ʹN, 113º24ʹE) and cultivated in the greenhouse at Henan Polytechnic University. The field collection followed the ethics and legality of the local government and was permitted by the government. Wei-Guo Li formally identified each sample, and the voucher specimens were deposited in the Herbarium of Wuhan Botanical Garden (HIB). Genomic DNA was extracted from fresh young leaves of sample SSM2 using the modified CTAB method for Illumina and High-Fidelity (HiFi) sequencing. A 350 bp insert library was constructed and sequenced for Illumina sequencing on a Novaseq6000 platform (Illumina, San Diego, California, USA). Approximately 70 Gb of clean sequence data with paired-end (PE) 150 reads were obtained and filtered using Fastp v0.12.6 [40] with default parameters. Additionally, a PacBio HiFi library with approximately 20 Kb fragments was prepared using the SMRTbell® Express Template Prep Kit 2.0 and sequenced on the PacBio Sequel platform at Beijing Novogene Bio Mdt InfoTech Ltd (Beijing, China). This generated approximately 27.9 Gb of HiFi CCS reads with an N50 length of 15,274 bp (Table S12). To improve the assembly at the chromosomal level, a Hi-C library was prepared from the same individual following standard protocols, including cell cross-linking, digestion, circularization, and DNA purification. High-quality DNA was digested using the restriction enzyme DPN II and then sequenced on the Novaseq6000 platform (Illumina, San Diego, California, USA). Approximately 114.6 Gb of clean data with PE150 reads were obtained (Table S12).

To predict gene models in the genome assembly, we collected and extracted total RNA from five tissues of sample SSM2, including root, stem, leaf, male and bisexual flowers, using the Plant Total RNA Isolation Kit (Sangon Biotech Co., Shanghai, China). Subsequently, cDNA libraries were constructed using the NEBNext Ultra RNA Library Prep Kit for Illumina (Illumina, NEB, USA) following the provided protocol. The libraries were then sequenced using the Illumina HiSeq XTen platform, generating approximately 6 Gb data (PE 150) for each sample.

### Genome size evaluation and genome assembly

To estimate the genome size of *T*. *rupestris* var. *ciliata* SSM2, we conducted *k*-mer frequency analysis on the Illumina data after filtering out plastid reads. The remaining reads were processed using Jellyfish v2.3.0 [41] to determine the *k*-mer distribution. Genome size and heterozygosity of SSM2 were evaluated using GCE v1.0.2 (ftp://ftp.genomics.org.cn/pub/gce). For de novo assembly, we employed Hifiasm v.0.19.4 [42] with default settings, and the resulting primary contigs were used to construct the chromosome-level assembly using Hi-C reads. By mapping clean Hi-C reads to the draft contig-level assembly with BWA v0.7.17 [43], we corrected contigs and scaffolded them with ALLHiC v0.9.13 [44] using recommended parameters. The interaction map was visualized and manually adjusted using JuiceBox v1.11.08 [45]. To assess the completeness and accuracy of the final genome assembly, we performed BUSCO (v4.0.1 [46]) analysis against the Embryophyta_odb10 database and utilized Merqury v1.3[47] for evaluation.

### Annotation of repeats, noncoding RNAs and protein-encoding genes

To identify repetitive elements in the genome assembly, we utilized TRF v4.09 [48] to detect tandem repeat sequences. For transposable element (TE) annotation, RepeatModeler v2.0.1 [49] was employed for de novo detection, and RepeatMasker v4.1.0 and RepeatProteinMask v3.3.0 (https://www.repeatmasker.org/) were used to identify known repeat sequences with the RepBase (v16.02) database. Also, EDTA v2.1.0 [50] was applied to identify and filter long terminal repeat retrotransposons (LTR-RTs) with default settings. The insertion time of intact LTR-RTs was estimated using LTR_retriever v2.9.0 [51], assuming a mutation rate of strawberries (2.8 × 10^–9^ /site/year [52]). To compare TE differentiation between cliff and non-cliff species in Rosaceae, we selected non-cliff species such as *Fragaria vesca*, *Prunus persica*, *Rosa chinensis*, *Malus domestica*, and *Cydonia sativa*. The same pipeline as above was employed to identify TEs and LTR-RTs in these species. TE density in gene and flanking regions was assessed using a sliding-window analysis with a window size of 100 bp and a step size of 20 bp. The RT domains of intact LTR-RTs were extracted, and a maximum-likelihood (ML) tree was constructed using Fasttree v2.1.11 [53]. After filtering redundant repeats in *T*. *rupestris* var. *ciliata*, the final repeat sequences were soft-masked for subsequent gene model prediction. In order to predict protein-coding genes (PCGs) in the genome assembly of *T*. *rupestris* var. *ciliata*, we employed three main strategies. Firstly, de novo prediction was performed using several software tools including Augustus v3.2.3[54], GlimmerHMM v3.0.4 [55], SNAP v2013.11.29 [56], Geneid v1.4 [57], and Genscan v1.0 [58], with default settings. Secondly, homolog prediction was accomplished by utilizing GeneWise v2.4.1 [59] and TblastN (v2.2.26; E-value ≤ 1e − 5 [60]) to match against homologous proteins from *F*. *vesca*, *R*. *chinensis*, *P*. *persica*, *Vitis vinifera*, and *Arabidopsis thaliana*. Lastly, for RNA-Seq-based prediction, all RNA-seq data from the five tissues were de novo and reference-guided assembled using Trinity v2.14.0 [61], and the PASA v2.4.1 pipeline [62] was employed to identify PCGs based on the assembled transcripts. To integrate all predicted gene models, EvidenceModeler v1.1.1 [63] was used, and subsequently, the annotations were updated by PASA, including modifications to exons, addition of UTRs, and incorporation of alternatively spliced models.

In order to annotate the predicted PCGs in the genome assembly of *T*. *rupestris* var. *ciliata*, we utilized several databases and tools. Firstly, for gene functional annotations, the PCGs were aligned against databases such as Swiss-Prot [64], KEGG (http://www.genome.jp/kegg/), NR (https://ftp.ncbi.nlm.nih.gov/), KOG (https://ftp.ncbi.nih.gov/pub/COG/KOG/), and GO [65]. This alignment was performed using diamond v2.1.0 [66] with e-value ≤ 1e − 5. Additionally, InterPro annotation was conducted using InterProScan v5.63–95.0 [67]. Furthermore, we employed Infernal v1.1.4 [68] to identify miRNAs and snRNAs by searching against the Rfam database [69], while tRNAs were annotated using tRNAscan-SE v1.3.1 [70] with default parameters. Lastly, the Blastn algorithm was employed to match against the Rfam database for rRNA identification.

### Genome evolution analysis

To perform a phylogenetic analysis and explore gene family evolution, we obtained protein-coding genes from 18 genomes, comprising two monocots, 14 dicots, and two basal angiosperms (Table S13). OrthoMCL v1.4 [71] was utilized to identify gene families among the 18 genomes, employing recommended settings. From this analysis, we identified 432 common single-copy orthologous genes, which were aligned using MAFFT v7.310 [72] with the L-INS-I strategy. The resulting alignments were then converted to codon sequences using PAL2NAL v14.1 [73]. To ensure alignment quality, we applied Gblocks v0.91 [74] to select conserved blocks from each alignment. These selected blocks were used to construct a ML tree using IQ-TREE v2.0.3 [75] with 5000 ultrafast bootstraps. Divergence time estimation among the 18 species was performed using MCMCtree [76]. After a burn-in of 1,500,000 iterations, the Markov chain Monte Carlo (MCMC) was run 50,000 times. We calibrated the divergence time using four reference points: the crown group of eudicots (125–161 Ma [77]), the crown group of Rosales (90–106.5 Ma [78]), the estimated divergence time between *Musa acuminata* and *Oryza sativa* ssp. *japonica* ranges from 103 to 117 Ma, and for *Solanum lycopersicum* and *Helianthus annuus*, *Acer truncatum* and *Arabidopsis thaliana*, they are between 97.5 to 109.2 Ma and 90 to 100.5 Ma, respectively, according to data from TimeTree (http://www.timetree.org/).. Furthermore, we investigated gene family expansion and contraction using CAFE v.4.2.1 [79]. Additionally, we performed GO and KEGG enrichment analysis on the expanded gene families using OmicShare tools (https://www.omicshare.com/tools).

To investigate the occurrence of whole genome duplication (WGD), we analyzed the distribution of synonymous substitutions (*Ks*) among paralogous genes in syntenic regions. Specifically, we compared the genomes of *F*. *vesca*, *P*. *persica*, and *V*. *vinifera* with *T*. *rupestris* var. *ciliata* to identify inter-genomic synteny. The *Ks* calculations and syntenic analyses were conducted using WGDI v0.5.7 [80] with default parameters, and extract event-related genomic alignment using Correspondence command in WGDI v0.5.7 [80] with recommended settings.

### Resequencing, SNP call, and demographic history

To compare the demographic history between *T*. *rupestris* var. *rupestris* and var. *ciliata* in Taihang Mountain, we sampled seven populations of var. *rupestris* and four populations of var. *ciliata* from across their entire distribution. One individual from each population was randomly selected for whole genome resequencing with an expected depth of over 25 × (Table S11). DNA extraction and library preparation followed the methods described for SSM2, and the sequencing was performed on the DNBSEQ-T7 platform (MGI Tech Co., Ltd., Guangdong, China) using the PE150 mode. The resulting clean reads were aligned to the *T. rupestris* var. *ciliata* genome using BWA v0.7.17 [43]. SAMtools v1.12 [81] was used to sort and merge the BAM files, and duplicate reads were removed with Picard v2.27 (http://broadinstitute.github.io/picard/). Variants, including SNPs and Indels, were identified using the GATK v4.2.0 package with default parameters [82]. Poor quality SNPs were dicarded in command VariantFiltration of GATK following: QD < 2.0 || MQ < 40.0 || FS > 60.0 || SOR > 3.0 || MQRankSum < -12.5 || ReadPosRankSum < -8.0. Biallelic loci were retained, and SNPs with a minor allele frequency (MAF) less than 0.01 and over 10% missing genotypes across individuals were excluded using VCFtools v0.1.16 [83]. Then, we further filtered sites with high LD to ensure the SNPs were unlinked with—- indep-pairwise parameter set as 50 10 0.2 using PLINK v1.90b6.21[84]. A total of 1,313,733 high-quality unlinked SNPs were used for population structure inference with ADMIXTURE v.1.3 [85], and a maximum likelihood (ML) tree was constructed using Fasttree v2.1.11 [53].

To infer the demographic history of *T*. *rupestris* var. *rupestris* and var. *ciliata*, we employed the PSMC v0.6.5 [86] to estimate the effective population size (*Ne*) based on deep-sequencing data from 11 individuals. The PSMC analysis was conducted with the following parameters: -N25 -t15 -r5 -p "4 + 25*2 + 4 + 6". The mutation rate of strawberries (2.8 × 10^–9^ /site/year [52]) was used, and we treated one generation as one year for both varieties.

### Comparative transcriptome analysis of leaves

To investigate the regulatory mechanisms underlying phenotypic variations in *T*. *rupestris*, we conducted a comparative analysis of mature leaves from the two varieties. Three biological replicates of each variety were collected from our common garden at Henan Polytechnic University. Total RNA was isolated, and RNA libraries were constructed following the previously described methods. The RNA-seq data, approximately 6 Gb per sample, were aligned to the genome assembly of *T*. *rupestris* var. *ciliata* using HISAT2 v2.2.1 [87] with default parameters. SAMtools v1.12 [81] was used to convert the aligned data to BAM format. Transcripts Per Million (TPM) and gene-level expression levels were quantified using featureCounts v1.6.0. [88] Differential expression analysis was performed using the edgeR v3.14 package [89], with the criteria of *P*-value < 0.05 and the logarithm of expression fold change (|logFC|> 1). The results were visualized using the EnhancedVolcano v1.18 package [90]. Additionally, GO and KEGG enrichment analyses were conducted using OmicShare tools (https://www.omicshare.com/tools) to gain insights into the functions and pathways associated with the differentially expressed genes (DEGs).

## Conclusions

In this study, we present the first chromosome-level genome assembly of *T*. *rupestris* var. *ciliata*, a cliff plant species. Our analysis explores the demographic history and molecular mechanisms underlying its adaptation to cliff habitats. The expansion of TE appears to have played a key role in the increase in genome size and the adaptation of *T*. *rupestris* var. *ciliata* to the challenging cliff environment. The effective population size of both varieties exhibited a continuous decline during the Quaternary climate changes, with no significant expansion after the Naynayxungla Glaciation. Limited seed dispersal and the absence of suitable habitats likely contributed to this pattern. Furthermore, the differential gene expression observed between the two varieties provides insights into their phenotypic leaf morphology variations and distinct responses to the harsh cliff conditions. It should be noted that variations in the copy numbers of gene families and their transcriptomic expression patterns may be associated with adaptability. Due to the limited genomic data available for cliff-dwelling plants, we have not compared differences in gene family copy numbers between cliff-dwelling and non-cliff-dwelling plants in the current study. In the future, it would be valuable to investigate the effects of gene family expansion on gene expression as more genomic data becomes available. Overall, our findings contribute to a better understanding of the demographic history and adaptive traits of *T*. *rupestris*, providing valuable insights into the evolution of cliff plants globally.

### Supplementary Information

Supplementary Material 1.

## Data Availability

All newly generated sequencing data have been deposited at the China National GeneBank DataBase (CNGBdb) under project accession No. CNP0004516. RNA-seq data are under accession No. CNX0759337-CNX0759347; resequencing data are under No. CNX0759349-CNX0759359. The HiFi, Hi-C, and genome survey reads are under accession No. CNX0759348, CNX0759335, and CNX0759336, respectively. The genome assembly and annotation files are available at 10.6084/m9.figshare.24448237.v2.

## Abbreviations

LTRs: Long terminal repeats
LTR-RTs: Long terminal repeat retrotransposons
MAF: Minor allele frequency
MCMC: Markov chain Monte Carlo
ML: Maximum-likelihood
PCGs: Protein-coding genes
TEs: Transposable elements
WGD: Whole genome duplication
