# Supplementary material for: Chromosome-level genome assembly of a cliff plant Taihangia rupestris var. ciliata provides insights into its adaptation and demographic history
Source: BMC Plant Biol. 2024 Jun 25;24:596. doi: 10.1186/s12870-024-05322-y (PMC11197248; doi:10.1186/s12870-024-05322-y)
Supplement: Supplementary file 1 — Supplementary Material 1. [file 12870_2024_5322_MOESM1_ESM.docx]

**Supplemetal Information for:**

**Chromosome-level genome** **assembly of a** **cliff plant** ***Taihangia rupestris* var. *ciliata* provides insights into its** **adaptation and demographic history**

Wei-Guo Li^1^*, Yuan-Yuan Li^1^, Chuan-Kun Zheng^1^, Zhi-Zhong Li^2^*

*^1^ School of Resource and Environment, Henan Polytechnic University, Jiaozuo, Henan 454000, China*

*^2^ Wuhan Botanical Garden, Chinese Academy of Sciences, Wuhan, China*

***Authors for correspondence:**

Wei-Guo Li, E-mail: wgli@hpu.edu.cn

Zhi-Zhong Li, E-mail: lizhizhong@wbgcas.cn

1. **Supplementary Figures**


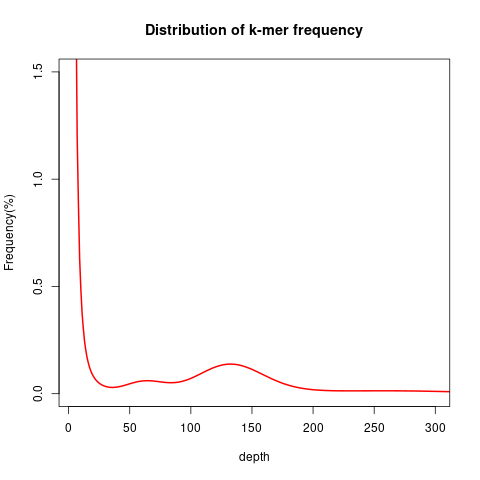


**Fig S1.** Distribution of 17-mer frequency for *Taihangia rupestris* var. *ciliata*


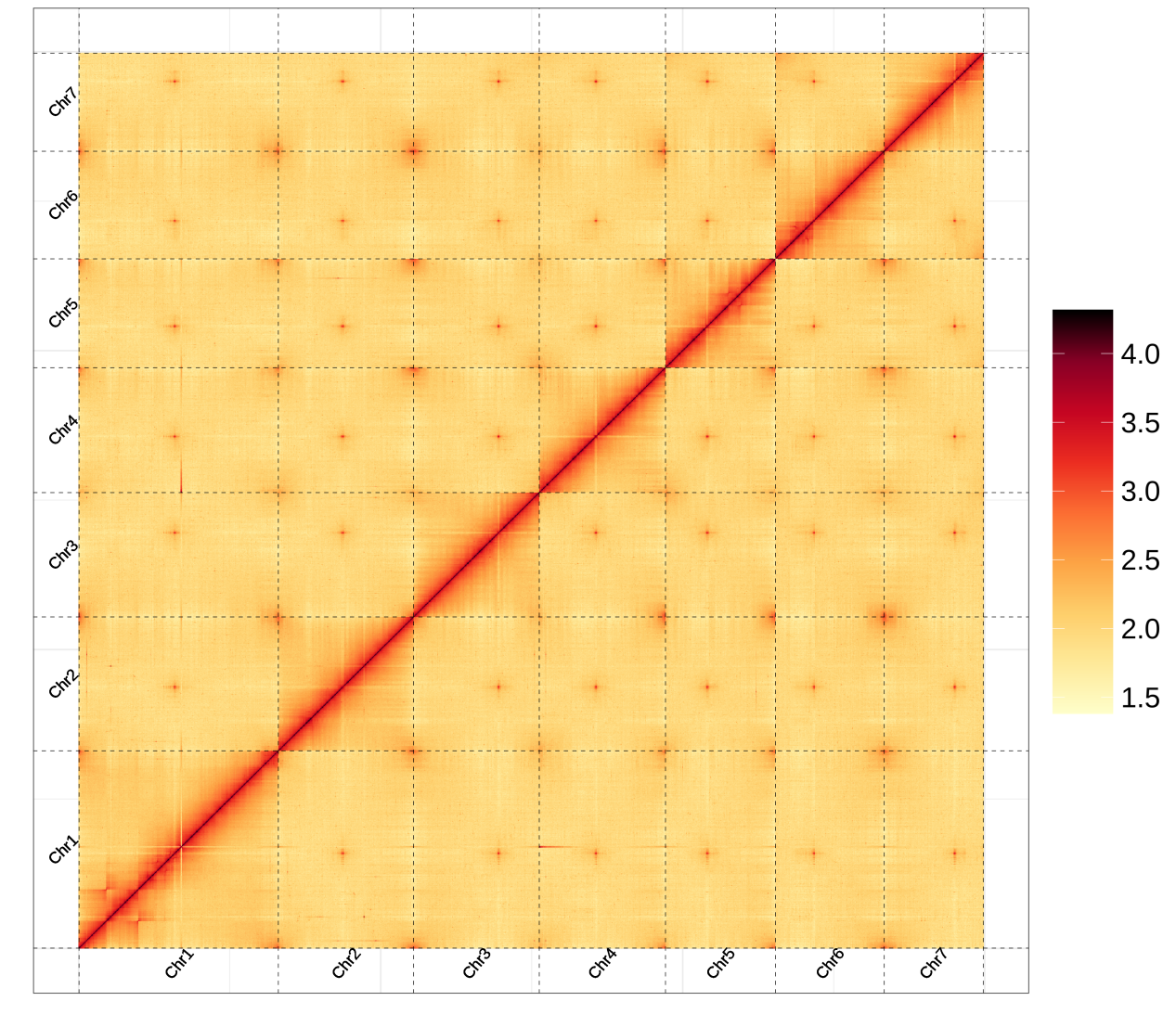


**Fig S2.** Heat map of the intensity for the Hi-C chromosome


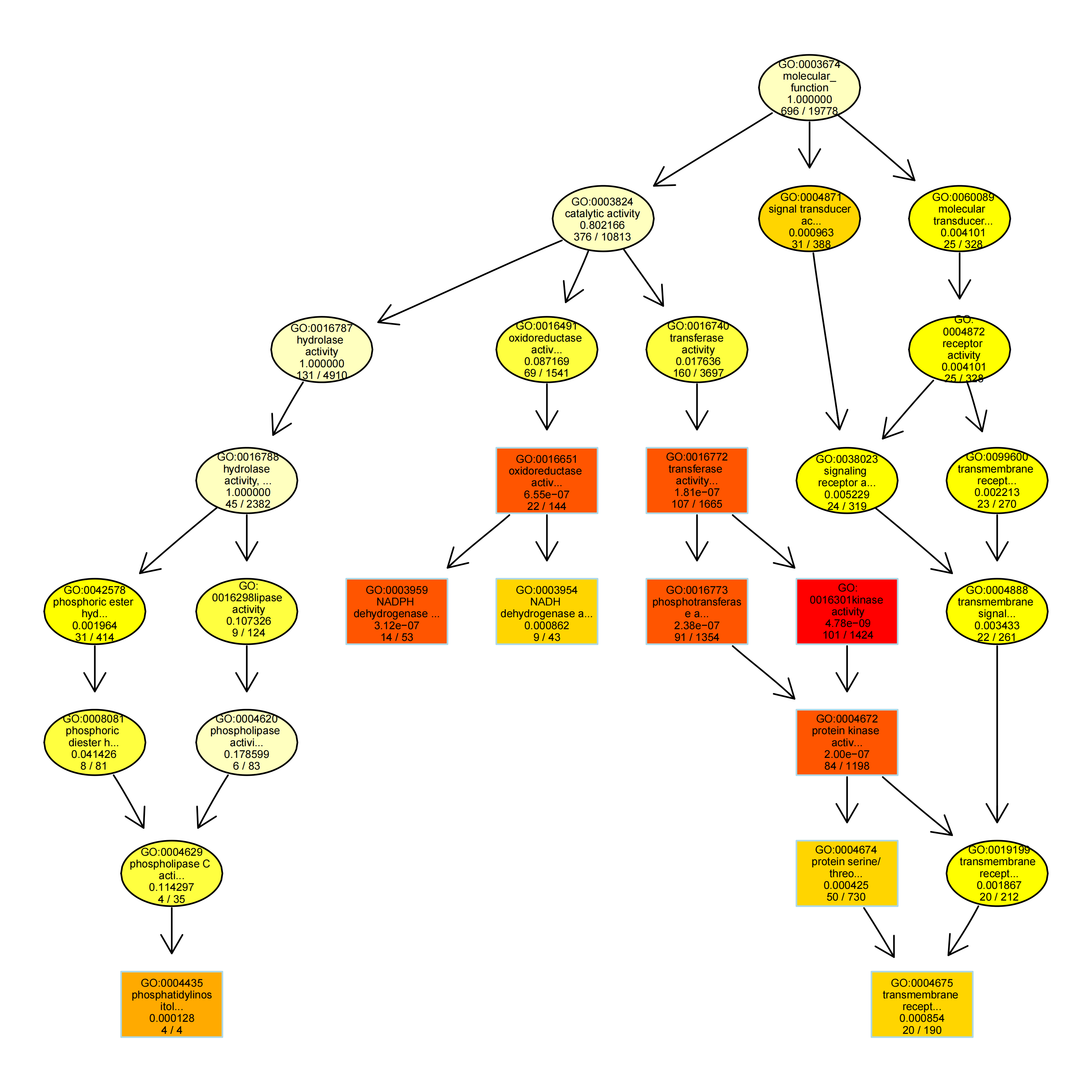


**Fig S3.** GO enrichment of *T. rupestris* var. *ciliata*-expanded genes


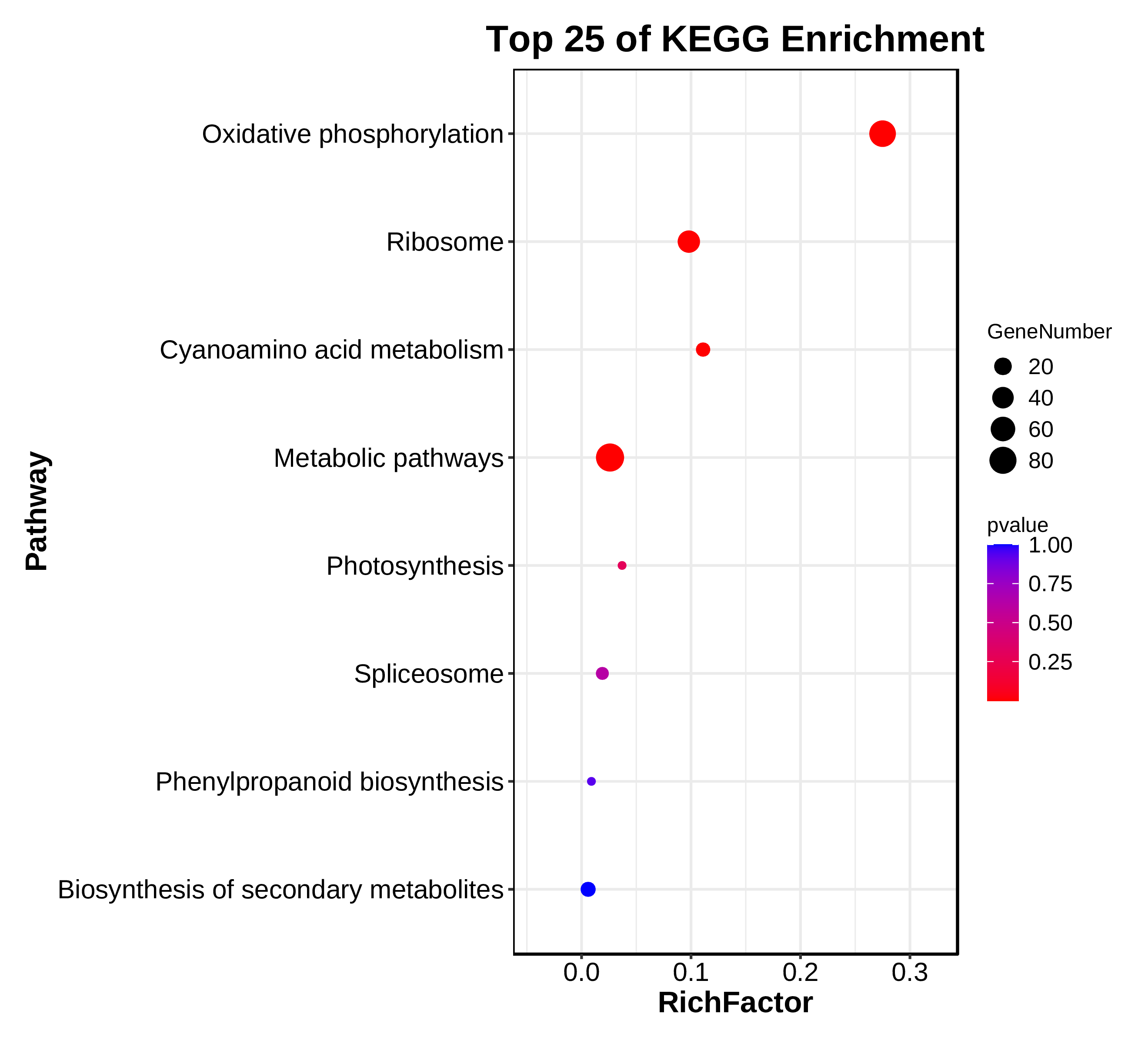


**Fig S4.** KEGG enrichment of *T. rupestris* var. *ciliata*-expanded genes


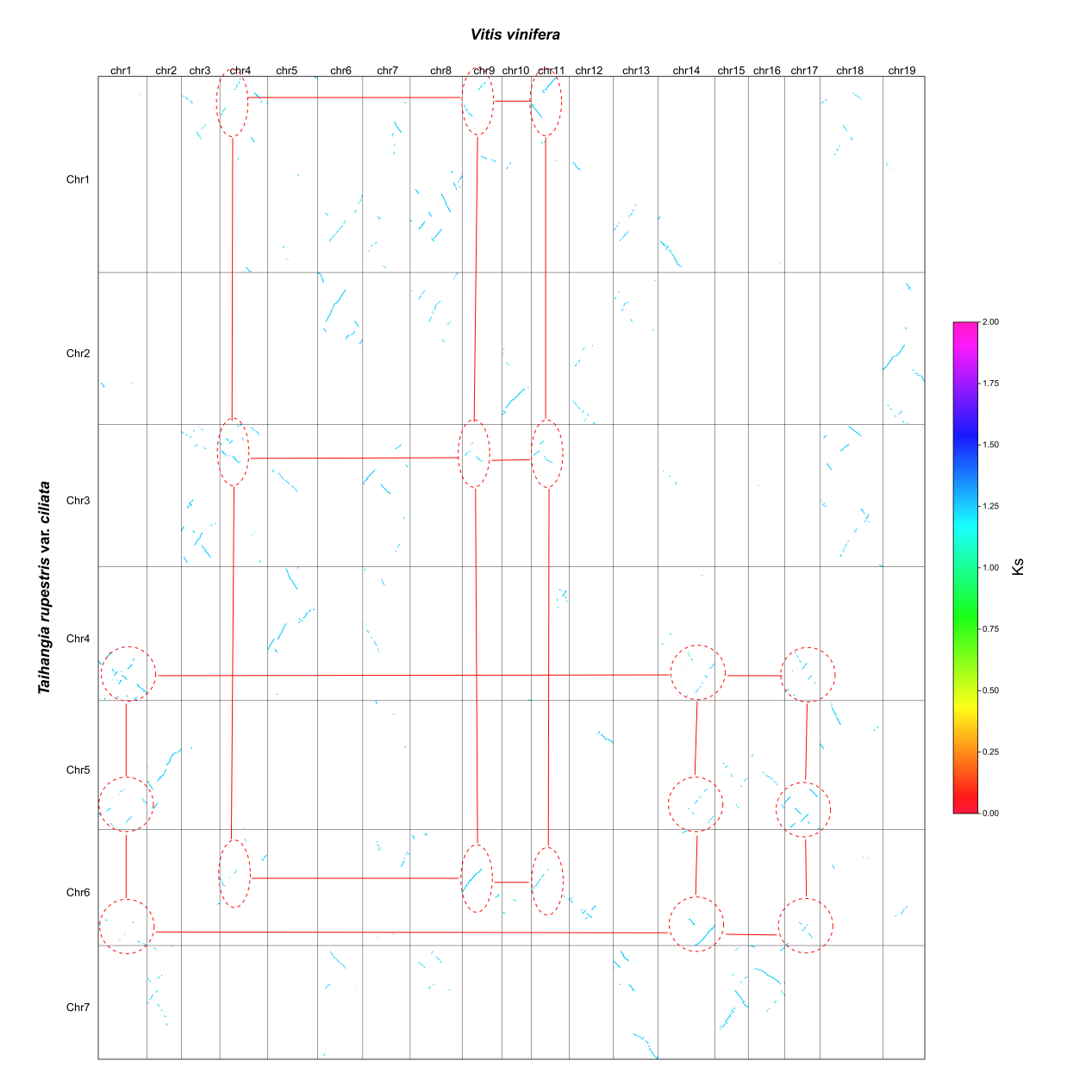


**Fig S5.** Syntenic analysis between *T. rupestris* var. *ciliata and V. vinifera*


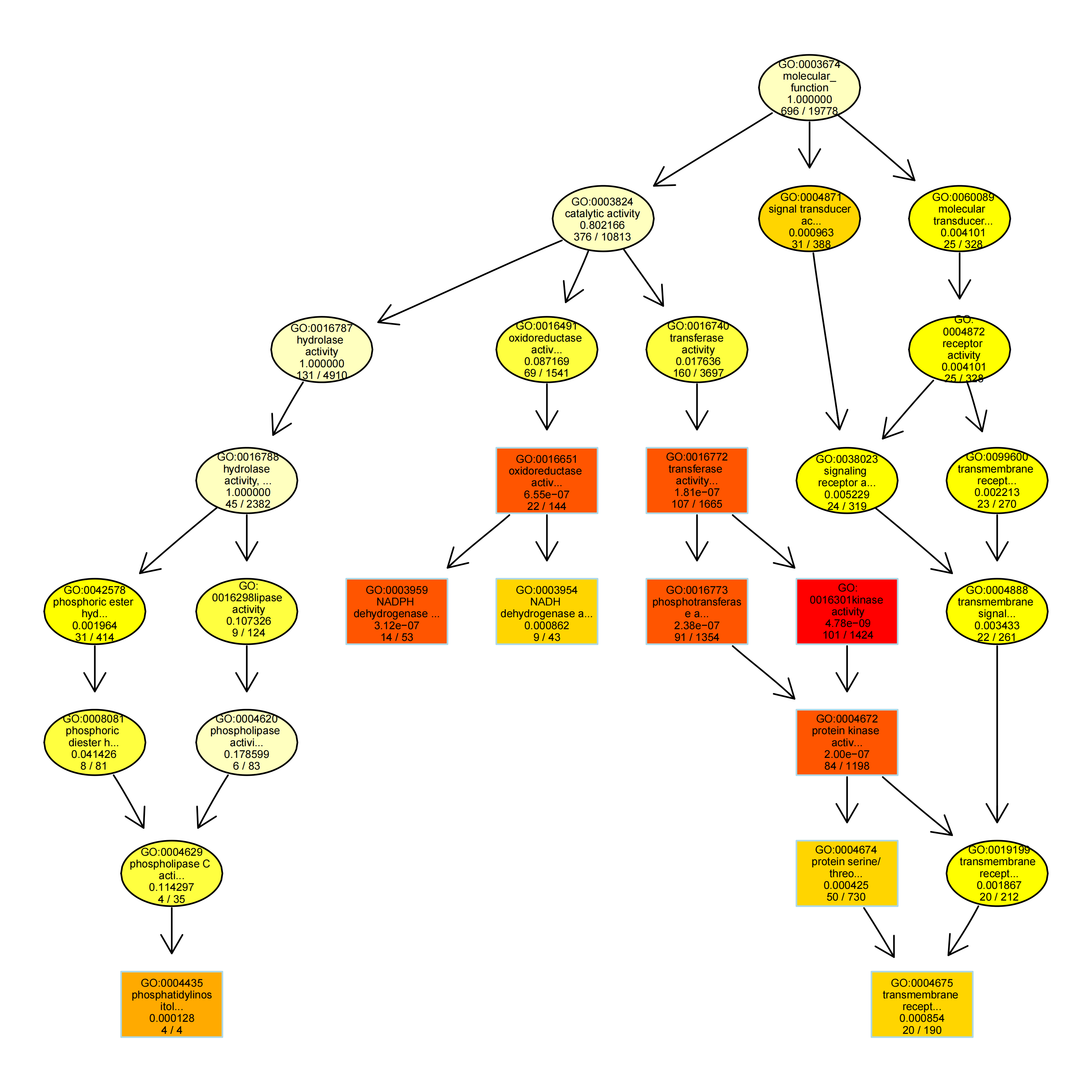
**Fig S6.** GO enrichment of WGT-related genes in *T. rupestris* var. c*iliata*


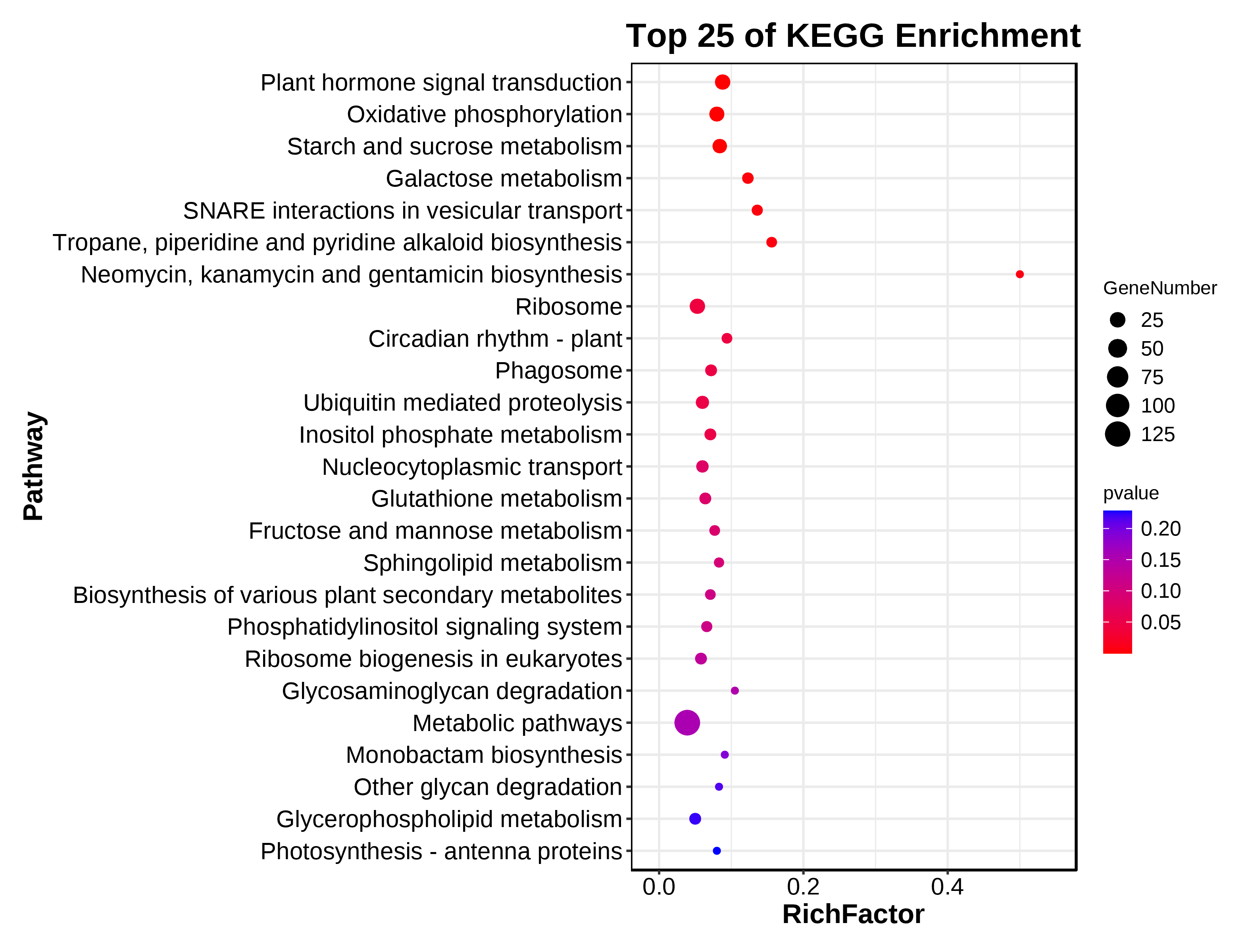


**Fig S7.** KEGG enrichment of WGT-related genes in *T. rupestris* var. *ciliata*

1. **Supplementary Tables**

**Table S1.** Statistics of the *T. rupestris* var. *ciliata* genome assembly

|  | Contig | | Scaffold* | |
| --- | --- | --- | --- | --- |
|  | Contig length | Contig number | Scaffold length | Scaffold number |
| Total | 769,501,930 | 309 | 769,508,730 | 241 |
| Longest | 49,193,729 | - | 165,965,130 | - |
| Number>=2000 | - | 309 | - | 241 |
| N50 | 17,869,299 | 13 | 104,920,189 | 4 |
| N60 | 15,829,046 | 18 | 104,920,189 | 4 |
| N70 | 12,821,381 | 23 | 91,187,168 | 5 |
| N80 | 10,112,734 | 30 | 90,489,742 | 6 |
| N90 | 5,928,420 | 40 | 82,670,173 | 7 |

* Only scaffolds with more than 100 bp length were used in genome assembly.

**Table S2.** Evaluation of the *T. rupestris* var. *ciliata* genome assembly using core Eukaryotic genes mapping approach (CEGMA)

| Category | Prot | Completeness (%) |
| --- | --- | --- |
| Complete | 236 | 95.16 |
| Complete + Partial | 241 | 97.18 |

**Table S3.** Evaluation of the *T. rupestris* var. *ciliata* genome assembly using Benchmarking Universal Single-Copy Orthologs (BUSCO)

| BUSCO assessment results (embryophyta_odb10, n = 1614*) | Percentage (%) |
| --- | --- |
| Complete BUSCOs | 98.3 |
| Complete and single-copy BUSCOs | 95.8 |
| Complete and duplicated BUSCOs | 2.5 |
| Fragmented BUSCOs | 0.2 |
| Missing BUSCOs | 1.5 |

* n: Total BUSCO groups searched

**Table S4.** Evaluation of the *T. rupestris* var. *ciliata* genome assembly using remapping of Illumina reads

|  |  | % of Percentage |
| --- | --- | --- |
| Reads | Mapping rate (%) | 99.52 |
| Genome | Average sequencing depth | 78.29 |
|  | Coverage ≥ 1× (%) | 99.98 |
|  | Coverage ≥ 4× (%) | 99.93 |
|  | Coverage ≥ 10× (%) | 99.85 |
|  | Coverage ≥ 20× (%) | 99.55 |

**Table S5.** Statistics of the *T. rupestris* var. *ciliata* and *T. rupestris* var. *rupestris* RNA-Seq data from different tissues

| Taxa | Tissues | Accession No. | Sequence Platform | Read Length (bp) | Clean data (G) | Q20/Q30 | Mapping to genome (%) |
| --- | --- | --- | --- | --- | --- | --- | --- |
| var. *ciliata* | root | CNR0866267 | Novoseq6000 | PE150 | 6.55 | 96.42/90.47 | 94.87 |
|  | stem | CNR0866268 |  |  | 6.75 | 96.74/91.37 | 94.14 |
|  | leaf | CNR0866269 |  |  | 6.09 | 96.55/90.82 | 94.26 |
|  | male flower | CNR0866270 |  |  | 6.64 | 96.55/90.79 | 93.28 |
|  | bisexual flower | CNR0866271 |  |  | 6.06 | 95.89/89.41 | 92.69 |
|  | mature Leaf (Tci1)* | CNR0866272 |  |  | 6.30 | 96.88/91.80 | 95.43 |
|  | mature Leaf (Tci2)* | CNR0866273 |  |  | 6.40 | 97.12/92.22 | 96.47 |
|  | mature Leaf (Tci3)* | CNR0866274 |  |  | 6.42 | 97.63/93.52 | 97.13 |
|  |  |  |  |  |  |  |  |
| var. *rupestris* | mature Leaf (Tru1)* | CNR0866275 |  |  | 6.20 | 96.75/91.52 | 93.65 |
|  | mature Leaf (Tru2)* | CNR0866276 |  |  | 6.00 | 96.92/91.70 | 94.07 |
|  | mature Leaf (Tru3)* | CNR0866277 |  |  | 6.48 | 97.68/93.59 | 94.61 |

* only used for comparative transcriptome analyses and other RNA-seq data was used for gene predict annotation in the *T. rupestris* var. *ciliata* genome assembly

**Table S6.** Summary of gene models in the *T. rupestris* var. *ciliata* genome assembly

| Predict strategy | Gene set | Number | Average transcript length (bp) | Average CDS length (bp) | Average exons per gene | Average exon length (bp) | Average intron length (bp) |
| --- | --- | --- | --- | --- | --- | --- | --- |
| *De novo* | Augustus | 47,057 | 1,789.52 | 951.38 | 3.71 | 256.27 | 309 |
|  | GlimmerHMM | 94,336 | 5,857.39 | 591.95 | 2.35 | 252.17 | 3,907.67 |
|  | SNAP | 39,547 | 8,254.24 | 502.2 | 4.11 | 122.15 | 2,491.51 |
|  | Geneid | 61,809 | 3,273.40 | 748.54 | 3.66 | 204.78 | 950.84 |
|  | Genscan | 49,100 | 10,151.01 | 1,214.26 | 5.88 | 206.61 | 1,832.40 |
| Homolog | *Fragaria vesca* | 30,719 | 2,293.03 | 1,024.20 | 4.04 | 253.68 | 417.74 |
|  | *Rosa chinensis* | 34,241 | 2,055.53 | 947.14 | 3.76 | 251.61 | 400.97 |
|  | *Arabidopsis thaliana* | 26,337 | 2,092.06 | 994.73 | 3.89 | 255.45 | 379.17 |
|  | *Prunus persica* | 28,431 | 2,328.89 | 1,060.77 | 4.17 | 254.67 | 400.64 |
|  | *Vitis vinifera* | 23,582 | 2,626.51 | 1,002.24 | 4.44 | 225.77 | 472.29 |
| RNAseq | PASA | 33,106 | 2,120.93 | 1,050.88 | 4.54 | 231.41 | 302.18 |
|  | Transcripts | 53,900 | 5,457.56 | 1,988.59 | 5.98 | 332.46 | 696.38 |
| **Final set** | | **36,300** | **2,563.92** | **1,058.99** | **4.37** | **242.48** | **446.91** |

**Table S7.** Summary of functional annotation for protein-coding genes in *T. rupestris* var. *ciliata*

| Database | Number | Percentage (%) |
| --- | --- | --- |
| Nr | 31,015 | 85.44 |
| KOG | 10,115 | 27.87 |
| Swissprot | 18,643 | 51.36 |
| GO | 22,584 | 62.21 |
| KEGG | 8,375 | 23.07 |
| InterPro | 28,522 | 78.57 |
|  |  |  |
| Total | 32,906 | 90.65 |

**Table S8.** Summary of non-coding RNA content in *T. rupestris var. ciliata*

| Type | | Copy number | Average length (bp) | Total length (bp) | % of genome |
| --- | --- | --- | --- | --- | --- |
| miRNA | | 591 | 137.2 | 81,086 | 0.010539 |
| tRNA | | 1584 | 74.8 | 118,483 | 0.015399 |
| rRNA | rRNA | 7513 | 372.52 | 2,798,727 | 0.36 |
|  | 18S | 1084 | 1736.49 | 1,882,360 | 0.24 |
|  | 28S | 4111 | 142.29 | 584,940 | 0.076023 |
|  | 5.8S | 1038 | 161.55 | 167,691 | 0.021794 |
|  | 5S | 1280 | 127.92 | 163,736 | 0.02128 |
| snRNA | snRNA | 2292 | 111.97 | 256,625 | 0.033353 |
|  | CD-box | 863 | 105.77 | 91,282 | 0.011864 |
|  | HACA-box | 62 | 131.48 | 8,152 | 0.001059 |
|  | splicing | 1366 | 114.89 | 156,945 | 0.020398 |
|  | scaRNA | 1 | 246 | 246 | 0.000032 |
|  | Unknown | 0 | 0 | 0 | 0 |

**Table S9**. Summary of transposable elements in the *T. rupestris* var. *ciliata* genome assembly

| Class |  | Count | Length (bp) | % in genome |
| --- | --- | --- | --- | --- |
| LTR |  |  |  |  |
|  | Copia | 84,255 | 74,766,505 | 9.72 |
|  | Gypsy | 115,441 | 172,639,711 | 22.44 |
|  | unknown | 192,322 | 154,552,309 | 20.09 |
| TIR |  |  |  |  |
|  | CACTA | 88,948 | 67,508,074 | 8.77 |
|  | Mutator | 63,883 | 22,334,287 | 2.9 |
|  | PIF_Harbinger | 33,992 | 14,487,289 | 1.88 |
|  | Tc1_Mariner | 1646 | 645,578 | 0.08 |
|  | hAT | 28,715 | 10,719,798 | 1.39 |
| nonTIR |  |  |  |  |
|  | helitron | 57,012 | 25,352,507 | 3.3 |
|  |  |  |  |  |
| Total |  | 666,214 | 543,006,058 | 70.57 |

**Table S10.** Comparison of transposon content in cliff and non-cliff plants in Rosaceae

|  | *T. rupestris* var. *ciliata* | *F. vesca* | *P. persica* | *R. chinensis* | *M. domestica* | *C. sativa* |
| --- | --- | --- | --- | --- | --- | --- |
| Habitat | cliff | non-cliff | non-cliff | non-cliff | non-cliff | non-cliff |
| Genome size (Mb) | 769 | 219 | 225 | 516 | 625 | 737 |
| TE content  (% in genome) | 543 Mb (70.57%) | 72.59 Mb  (33.08%) | 73.84 Mb (32.87%) | 290.37 Mb (56.32%) | 356.79 Mb (57.10%) | 504.52 Mb (68.50%) |
| Number of Intact LTR-RTs  (*Copia*/*Gypsy*) | 8558  (3729/4829) | 1098  (683/415) | 1132  (804/328) | 6033  (4265/1768) | 5421  (3251/2170) | 7226  (4698/2528) |

**Table S11.** Summary of resequencing analysis

| Taxa | Individuals | Localities | Latitude | Longitude | Resource type | Clean data (Gb) | Sequencing depth (×)* | Accession No. |
| --- | --- | --- | --- | --- | --- | --- | --- | --- |
| var. *ciliata* | JG-1 | Jiaogou, Hebei | 36º30ʹ | 113°38ʹ | PE150 bp,  DNBSEQ-T7 | 24.91 | 28.86 | CNR0866279 |
|  | QQ-2 | Qingquan, Shanxi | 36º44ʹ | 113°32ʹ |  | 21.57 | 24.99 | CNR0866280 |
|  | DJC-2 | Dujiacun, Hebei | 36º53ʹ | 113°47ʹ |  | 21.6 | 25.02 | CNR0866281 |
|  | LJ-3 | Liejiang, Hebei | 36º54ʹ | 113°46ʹ |  | 26.18 | 30.33 | CNR0866282 |
| var. *rupestris* | MZ-1 | Manzhanshan, Henan | 36°05ʹ | 113°42ʹ |  | 21.62 | 25.04 | CNR0866283 |
|  | XXT-1 | Xiaoxitian, Henan | 36°03ʹ | 113°40ʹ |  | 21 | 24.33 | CNR0866284 |
|  | QPG-1 | Qianpogou, Henan | 36°02ʹ | 113°39ʹ |  | 26.34 | 30.51 | CNR0866285 |
|  | YDS-1 | Yidoushui, Henan | 35°28ʹ | 113°23ʹ |  | 21.8 | 25.25 | CNR0866286 |
|  | ZYF-1 | Zhuyufeng, Henan | 35°27ʹ | 113°22ʹ |  | 19.37 | 22.44 | CNR0866287 |
|  | GS-1 | Hongshixia,Henan | 35°33ʹ | 113°30ʹ |  | 25.31 | 29.32 | CNR0866288 |
|  | TZF-1 | Tianzhufeng,Henan | 35°33ʹ | 113°31ʹ |  | 21.12 | 24.47 | CNR0866289 |

*Sequencing depth was estimated according to the estimated genome size of 863.27M

**Table S12.** Genome sequencing information for *Taihangia rupestris* var. *ciliata*

| Library type | Clean data (Gb) | Read N50/ length (bp) | Sequencing depth (×)* |
| --- | --- | --- | --- |
| Illumina (Genome survey) | 70 | 150 | 81 |
| Illumina (Hi-C) | 114.6 | 150 | 132 |
| HiFi | 27.9 | 15,274 | 32 |
| Total | 212.5 |  | 245 |

*Sequencing depth was estimated according to the estimated genome size of 863.27M

**Table S13.** Genomic resources used for comparative analyses in this study

| Species name | Database link |
| --- | --- |
| *Amborella trichopoda* | https://www.ncbi.nlm.nih.gov/datasets/genome/GCF_000471905.2/ |
| *Arabidopsis thaliana* | https://bioinformatics.psb.ugent.be/plaza/versions/plaza_v5_dicots/organism/view/ath |
| *Populus trichocarpa* | https://bioinformatics.psb.ugent.be/plaza/versions/plaza_v5_dicots/organism/view/ptr |
| *Davidia involucrata* | https://bioinformatics.psb.ugent.be/plaza/versions/plaza_v5_dicots/organism/view/din |
| *Solanum lycopersicum* | https://bioinformatics.psb.ugent.be/plaza/versions/plaza_v5_dicots/organism/view/sly |
| *Helianthus annuus* | https://bioinformatics.psb.ugent.be/plaza/versions/plaza_v5_dicots/organism/view/han |
| *Nelumbo nucifera* | http://nelumbo.cngb.org/downloadData/download?path=NNU.genomic.fa |
| *Fragaria vesca* | https://bioinformatics.psb.ugent.be/plaza/versions/plaza_v5_dicots/organism/view/fve |
| *Rosa chinensis* | https://bioinformatics.psb.ugent.be/plaza/versions/plaza_v5_dicots/organism/view/rch |
| *Malus domestica* | https://bioinformatics.psb.ugent.be/plaza/versions/plaza_v5_dicots/organism/view/mdo |
| *Prunus persica* | https://bioinformatics.psb.ugent.be/plaza/versions/plaza_v5_dicots/organism/view/ppe |
| *Cannabis sativa* | https://bioinformatics.psb.ugent.be/plaza/versions/plaza_v5_dicots/organism/view/cansat |
| *Vitis vinifera* | https://bioinformatics.psb.ugent.be/plaza/versions/plaza_v5_dicots/organism/view/vvi |
| *Acer truncatum* | https://bioinformatics.psb.ugent.be/plaza/versions/plaza_v5_dicots/organism/view/acertr |
| *Musa acuminata* | https://bioinformatics.psb.ugent.be/plaza/versions/plaza_v5_monocots/organism/view/mac |
| *Oryza sativa* ssp. *j*aponica | https://bioinformatics.psb.ugent.be/plaza/versions/plaza_v5_monocots/organism/view/osa |
| *Nymphaea colorata* | https://www.ncbi.nlm.nih.gov/datasets/genome/GCF_008831285.2/ |
